# Improved Plaque-Induced Gingivitis in Students Using Calibrated Interdental Brushes: Results of a 3-Month Multicenter Educational Intervention

**DOI:** 10.3390/jcm14165738

**Published:** 2025-08-14

**Authors:** Marta Mazur, Flavia Vitiello, Artnora Ndokaj, Rossana Izzetti, Vincenzo Tosco, Denise Corridore, Chiara Mannucci, Riccardo Monterubbianesi, Maria Rita Giuca, Livia Ottolenghi, Giovanna Orsini, Florence Carrouel, Denis Bourgeois

**Affiliations:** 1 Department of Oral and Maxillofacial Sciences, Sapienza University of Rome, 00161 Rome, Italy; marta.mazur@uniroma1.it (M.M.); artnora.ndokaj@uniroma1.it (A.N.); livia.ottolenghi@uniroma1.it (L.O.); 2 Department DISCO, Università Politecnica Delle Marche, 60126 Ancona, Italy; f.vitiello@pm.univpm.it (F.V.); v.tosco@pm.univpm.it (V.T.); r.monterubbianesi@staff.univpm.it (R.M.); g.orsini@staff.univpm.it (G.O.); 3 Unit of Oral Medicine and Oral Surgery, School of Medicine and Surgery, University of Pisa, 56126 Pisa, Italy; rossana.izzetti@unipi.it (R.I.); mannucci.chiara@libero.it (C.M.); mariarita.giuca@unipi.it (M.R.G.); 4 Unit of Oral Medicine and Oral Surgery, School of Medicine and Surgery, Link Campus University, 00165 Rome, Italy; denise.corridore@gmail.com; 5 Laboratory “Health Systemic Process" (P2S), UR4129, University Claude Bernard Lyon 1, University of Lyon, 69008 Lyon, France; florence.carrouel@univ-lyon1.fr

**Keywords:** interdental brushing, gingival bleeding, preventive oral care, oral health education, oral hygiene practices, dental students

## Abstract

**Objective:** To evaluate the short-term clinical impact of daily use of calibrated interdental brushes (IDBs) on gingival bleeding among dental and dental hygiene students within academic curricula. **Methods:** A prospective cohort of 117 students from three Italian universities was followed over three months. All participants received personalized training and calibrated interdental brushes matched to their interdental spaces. The primary outcome was the percentage of interdental sites exhibiting bleeding on interdental brushing (BOIB), assessed at baseline (T0), one month (T1), and three months (T2). Adherence was self-reported. Statistical analyses included Wilcoxon tests, multivariate regression, and adjusted ANCOVA models. **Results:** Median bleeding scores decreased from 50.0% [IQR: 26.9–69.2] at baseline to 15.4% [IQR: 3.8–30.8] at one month and further to 11.5% [IQR: 0.0–26.9] at three months (*p* < 0.001). Regular interdental brush users showed a 15 to 16 percentage point greater reduction in bleeding compared to occasional users (*p* < 0.001). Dental hygiene students had significantly lower baseline bleeding scores than dental students, but both groups experienced comparable benefits from the intervention. Adjusted analyses confirmed a sustained beneficial effect of regular interdental brushing. Initial weak and transient correlations between behavioral factors and bleeding likely reflect multifactorial influences and variable adherence. **Conclusions:** Daily use of calibrated interdental brushes produces a rapid, significant, and sustained reduction in gingival bleeding among dental students. Systematic integration of this protocol within dental education programs is feasible and effective, promoting early adoption and maintenance of essential preventive oral health behaviors.

## 1. Introduction

Maintenance of periodontal health is a cornerstone of preventive dentistry and is fundamentally linked to the effective disruption of oral biofilm [1,2]. While daily toothbrushing represents the primary strategy to control supragingival plaque, this approach remains inadequate for accessing interdental spaces, ecological niches that harbor complex and potentially pathogenic biofilm communities [3]. Evidence has shown that interdental biofilm may contribute not only to local inflammatory conditions, such as gingivitis and periodontitis, but may also act as a reservoir for systemic pathogens, posing broader health risks, including cardiovascular and metabolic diseases [4].

IDBs have emerged as the most effective mechanical tool for disrupting biofilm in accessible interdental spaces [5]. Historically, IDBs were recommended mainly for periodontally compromised patients presenting with open embrasures [6]. However, research increasingly supports their broader application, even among periodontally healthy individuals [7]. Notably, calibrated interdental brushes have been demonstrated to significantly reduce interdental bleeding, a clinical hallmark of gingival inflammation, when used daily, with the effect observed as early as one week and sustained over three months [8].

Despite these clinical benefits, interdental cleaning remains underutilized in routine oral hygiene, particularly among younger populations [9]. One of the main barriers remains educational. A previous investigation of a dedicated interdental prophylaxis teaching module in dental students showed positive changes in attitudes and behaviors towards the use of IDBs, although adherence and long-term integration into daily routines remained suboptimal [10]. These findings support the importance of early and structured education in interdental cleaning to empower future dental professionals and their patients.

Beyond local effects on oral health, the interdental microbiota plays an increasingly recognized role in systemic health [11,12]. The interdental biofilm harbors a variety of pathogens, including Streptococcus mutans, Candida albicans, and periodontopathogens from the “red complex”, which are associated not only with periodontal disease but also with systemic conditions such as cardiovascular disease and inflammatory bowel diseases [4,13,14,15]. Disruption of the oral microbial balance, particularly in interdental areas often missed by routine brushing, can lead to dysbiosis, which has been implicated in systemic inflammatory responses [16]. Thus, strategies aimed at maintaining interdental biofilm control, particularly through calibrated educational approaches among young dental professionals, are critical not only for preventing oral diseases but also for contributing to overall health homeostasis [3,17].

In light of this background, and recognizing that dental students and dental hygiene students represent the oral health professionals of tomorrow, targeted interventions are needed to evaluate the impact of calibrated interdental cleaning on periodontal parameters, especially bleeding on interdental brushing, which may serve as a motivational indicator to enhance compliance [18]. Therefore, the present prospective cohort study aimed to investigate the effect of calibrated interdental brush use on gingival bleeding in dental and dental hygiene students over a three-month period. By combining educational and clinical outcomes, this study aims to bridge the gap between knowledge acquisition and behavioral implementation of interdental cleaning in daily oral hygiene routines.

This study aimed to evaluate the short-term impact of calibrated interdental brushing on gingival bleeding among dental and dental hygiene students engaged in structured oral health education.

## 2. Materials and Methods

This manuscript was conducted in accordance with the STROBE (Strengthening the Reporting of Observational Studies in Epidemiology) guidelines for observational cohort studies. This study was conducted in collaboration with three Italian academic institutions: Università Politecnica delle Marche, Sapienza Università di Roma, and Università di Pisa. The research protocol received ethical approval from the respective local Ethics Committees at each site (Sapienza approval no. 0399; Università Politecnica delle Marche approval no. 0074624; Università di Pisa approval no. 00052024). Written informed consent was obtained from all participants prior to enrollment. All procedures were carried out in accordance with the ethical principles of the Declaration of Helsinki (1964) and its subsequent amendments, or with comparable institutional and international ethical standards.

### 2.1. Study Design

This was a prospective cohort study conducted over a three-month period to evaluate the clinical impact of calibrated interdental brushing on gingival bleeding. The study was intentionally embedded in the educational environment of second-year dental and dental hygiene students, using a pragmatic approach. The clinical protocol was incorporated into existing teaching modules with minimal disruption to the core curriculum. This real-world integration aimed to assess the effectiveness of a structured interdental brushing protocol when implemented through institutional academic frameworks.

### 2.2. Study Setting and Population

The study involved the entire cohort of second-year dental and dental hygiene students enrolled during the 2023–2024 academic year at the three participating universities. This approach ensured full coverage of the target population and alignment with the pragmatic design. Both theoretical and clinical components were included, and follow-up assessments were scheduled as part of the academic calendar.

### 2.3. Objectives

The primary objective was to evaluate the short-term clinical impact of an individually calibrated interdental brushing protocol on gingival bleeding. A secondary objective was to document preliminary insights into the integration of the protocol within existing academic teaching structures.

### 2.4. Inclusion and Exclusion Criteria

Eligible participants were (i) second-year dental or dental hygiene students, (ii) aged 18 years or older, and (iii) provided informed consent. Exclusion criteria included (i) students undergoing any periodontal or orthodontic therapy, (ii) the presence of systemic diseases affecting periodontal status, or (iii) antibiotic use in the previous three months.

### 2.5. Ethical Approval and Consent to Participate

Participation in the study was voluntary, and no incentives (financial or academic) were offered. Students received verbal and written information describing the research objectives, procedures, and data privacy protections. While the educational module was a required part of the curriculum, participation in the clinical data collection (bleeding scores at T0, T1, and T2) was optional. Students were explicitly informed that declining participation would have no impact on their course grades or academic evaluation.

Importantly, some students declined to participate in follow-up visits, including for cultural or religious reasons (e.g., Ramadan), confirming that participation was genuinely voluntary. Clinical assessments were conducted by trained investigators who were not involved in course grading, ensuring independence from any educational evaluation process.

### 2.6. Educational Framework

**Figure 1:**
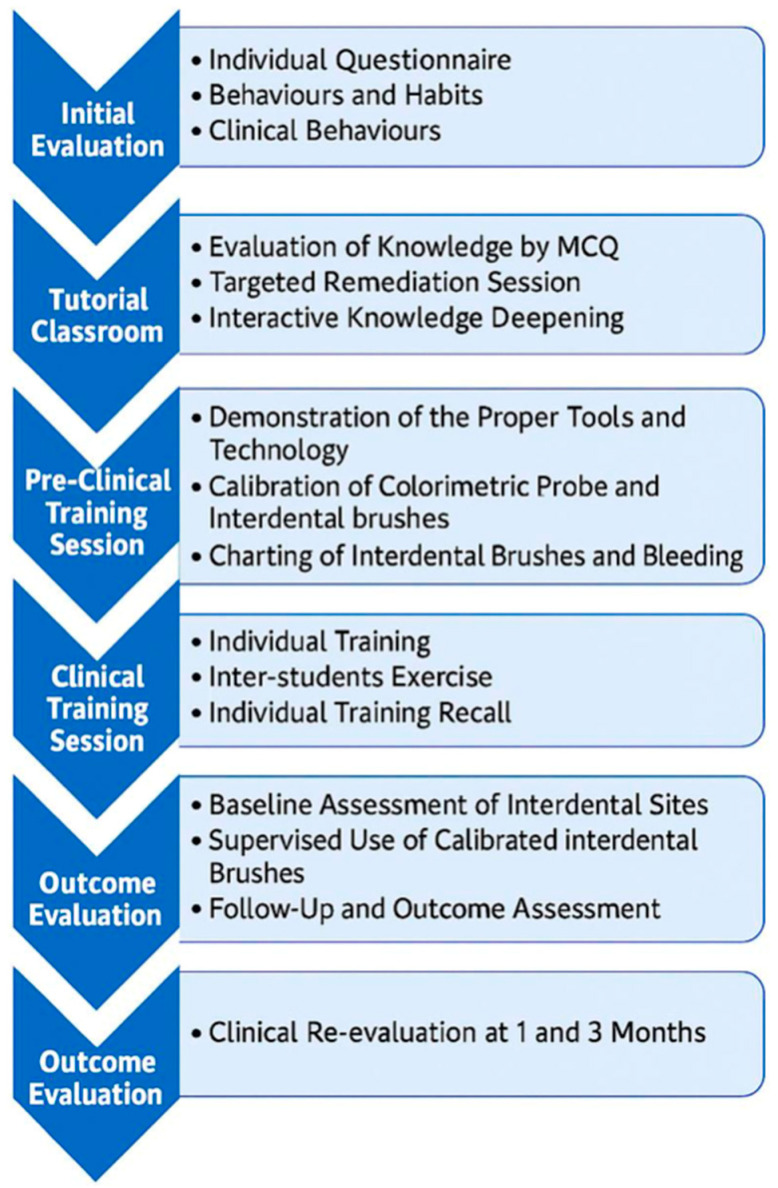
Workflow of the evaluation: six-step educational sequence for interdental prophylaxis training. Adapted from: Bourgeois D, et al. [10].

To structure the acquisition and long-term retention of interdental prophylaxis competencies, a six-phase educational sequence was implemented. This model integrates theoretical knowledge, preclinical skill development, supervised clinical practice, and outcome-based evaluation. Each phase builds upon the previous one, reinforcing both behavioral change and clinical efficacy. The entire workflow is summarized in Figure 1.

This structured model includes initial assessment, tutorial instruction, preclinical training, clinical implementation, supervised clinical phase, and longitudinal outcome evaluation. Each step progressively supports students in acquiring, applying, and sustaining interdental hygiene practices.

In Supplementary files, Tables S1–S3 present a detailed synthesis of the six-phase educational sequence implemented in this study. In contrast to conventional curricular descriptions, this table articulates the specific learning objectives, instructional content, and delivery format for each phase. By aligning these three dimensions, the table provides a clear mapping of how the program operationalizes the transition from theoretical knowledge to clinical proficiency. This pedagogical structuring reflects a deliberate effort to promote standardization across institutions, reinforce technical autonomy, and ensure the reproducibility of instructor-led interventions. It also supports the longitudinal integration of behavioral change through a stepwise progression of supervised and autonomous practice.

### 2.7. Clinical Assessments and Follow-Up

Clinical examinations were performed at baseline (T0), one month (T1), and three months (T2). At each time point, the percentage of interdental sites exhibiting bleeding on interdental brushing (BOIB) was recorded. Baseline characteristics included age, sex, smoking status, type of toothbrush, brushing frequency, self-reported bleeding, and frequency of interdental brush use. Data were recorded using a standardized electronic charting system. Follow-up adherence was supported by scheduled appointments and electronic reminders.

### 2.8. Interdental Brushing Protocol

At baseline, interdental space assessment was performed using the IAP Curaprox© color-coded probe (Curaden, Kriens, Switzerland), which enabled precise sizing. Each participant was assigned one or more calibrated interdental brushes (Curaprox© CPS; Curaden) corresponding to the measured dimensions. Initial use of the brushes took place under supervision by qualified public health faculty. Participants received both verbal and practical instruction on correct technique, without additional oral hygiene training. They were instructed to use the assigned brushes daily, following manufacturer guidelines, while maintaining their usual oral hygiene practices. No professional periodontal treatment was administered during the study.

### 2.9. Sample Size

A convenience sample of 117 students was included, based on class size and logistical feasibility. No formal power calculation was performed, as the study was exploratory. The chosen sample size was deemed sufficient to detect meaningful clinical changes based on previous studies involving interdental brushing in similar populations [10].

### 2.10. Educational Standardization and Instructor Training

The same team of instructors, drawn from the three participating universities, delivered the intervention uniformly across all sites. Prior to the start of the study, these instructors completed a 16-h standardized theoretical and practical training program. This calibration ensured consistent delivery of teaching content, clinical supervision, and assessment procedures. A single set of standardized presentation materials, including slides, was used across all centers.

Calibrated instructors were responsible for performing all procedures involving the classification of interdental access diameter, the verification of booster effectiveness (i.e., the match between probe-based sizing and brush diameter), and the documentation of bleeding scores. These activities were conducted consistently at all sites and at all phases, prior to the delivery of clinical instruction to students.

Seven trained investigators performed the gingival bleeding assessments across the three academic centers: two in Pisa (MC, RI), two in Rome (MM, DC), and three in Ancona (FV, VT, RM). All examiners were experienced clinicians affiliated with the participating universities. Prior to study initiation, all investigators participated in a centralized training and calibration program. This included a 6-h theoretical module (delivered remotely), followed by a 2-day in-person seminar led by a senior periodontist (DB). Duplicate examinations were conducted on 20 volunteer students to assess inter-examiner reliability.

During the central calibration session, each examiner evaluated four volunteer subjects (112 interdental sites) using the IAP Curaprox© color-coded probe (Curaden, Kriens, Switzerland), which displays five distinct color bands corresponding to brush sizes. The assessment criterion was the identification of the correct color score per site, compared with a gold standard examiner (DB). Site-level agreement with the reference examiner reached at least 85% for all participants, indicating satisfactory reproducibility.

Following this session, baseline and follow-up clinical assessments were conducted by these calibrated examiners across the three academic centers, under supervision (DB), ensuring standardized data collection throughout the study.

### 2.11. Clinical Procedures

All clinical procedures were conducted under standardized conditions and supervised by faculty members specialized in public health and preventive dentistry. At baseline, each student underwent a full interproximal examination performed using the IAP colorimetric probe (Curaprox, Kriens, Switzerland). This device allowed for the classification of interdental space diameter, used to determine the appropriate size of the calibrated interdental brush (Curaprox CPS).

Students were then instructed to perform interdental brushing using the assigned CPS brushes under direct supervision. Instructors provided correction of insertion angle, pressure, and movement technique when necessary. The Touch-to-Teach approach was adopted during this session: each student alternated roles between operator, patient, and observer, enhancing tactile understanding of interdental access and bleeding control.

At each interproximal site, students recorded:

- the access diameter, based on IAP color code,
- and the bleeding response observed upon brushing (BOIB).

These data were documented using a standardized clinical charting form. Re-assessments were scheduled at 1 month (T1) and 3 months (T2), during which students repeated the same procedures. No additional professional prophylactic treatment was administered during the study period.

### 2.12. Statistical Analysis

To investigate changes in gingival bleeding over time and assess the influence of clinical and behavioral factors, we implemented a multivariable statistical strategy combining exploratory correlations and adjusted longitudinal models. Descriptive statistics were used to summarize baseline characteristics. Normality of bleeding scores at each visit was tested using the Shapiro–Wilk test and confirmed to be non-Gaussian (*p* < 0.001), supporting the use of non-parametric methods. To explore associations between gingival bleeding and clinical or behavioral variables at each time point (T0, T1, T2), Spearman’s rank correlation coefficients were calculated. For longitudinal comparisons of bleeding scores, we implemented Generalized Estimating Equations (GEE), which are appropriate for non-parametric repeated measures data with within-subject correlation. The GEE model included interdental brush usage group, time, and their interaction and was adjusted for relevant covariates: baseline bleeding (T0), age, sex, smoking status, toothbrush type, educational group (dental vs. hygiene students), and study center (Rome, Pisa, Ancona).

This modeling approach allowed us to estimate adjusted mean bleeding scores over time and to test group differences while accounting for individual variability and institutional context. Statistical analyses were conducted using R (version 4.4.0). A two-sided *p*-value < 0.05 was considered statistically significant.

## 3. Results

### 3.1. Baseline Characteristics

At baseline (T0), 117 participants were included in the analysis. The mean age was 22.8 years (SD = 2.3), and the average percentage of bleeding sites was 32.5% (SD = 17.4). The majority of participants were female (77.8%) and non-smokers (86.3%). Most reported using manual toothbrushes (54.7%), while the remainder used electric toothbrushes.

In terms of oral hygiene behaviors, 55.6% of participants reported brushing their teeth two to three times per day, 26.5% brushed more than three times per day, and 17.9% brushed less than twice daily. Regarding interdental cleaning habits, 94.9% reported at least occasional use of interdental brushes (IDBs), with 23.1% indicating frequent use.

Self-reported gingival bleeding was noted by 58.1% of participants. The study sample comprised 66.7% dental students (DS) and 33.3% dental hygiene students (DH).

### 3.2. Evolution of Gingival Bleeding over Time

**Figure 2:**
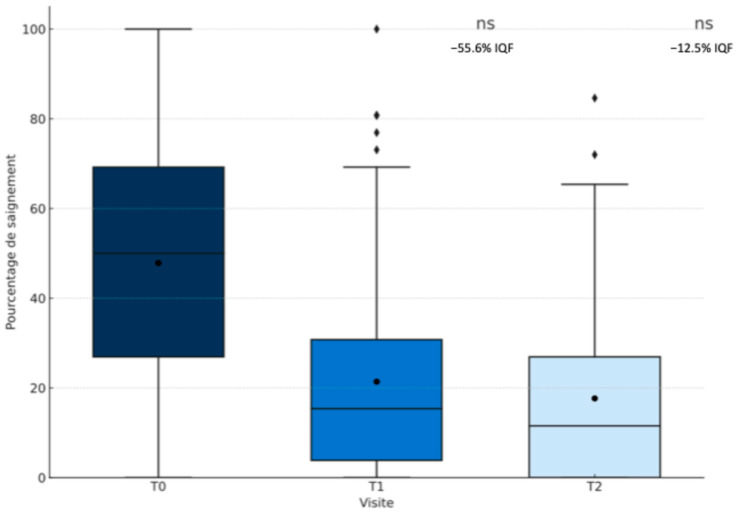
Reduction in the percentage of bleeding sites per subject across clinical visits (T0, T1, T2).

Over the course of the study, a marked and consistent reduction in the percentage of bleeding sites per subject was observed. As shown in Figure 2, at baseline (T0), the median bleeding score was 50.0%, with an interquartile range (IQR) of 26.9% to 69.2%, reflecting a relatively high and variable level of gingival inflammation among participants. One month later (T1), this median value had decreased substantially to 15.4% (IQR: 3.8–30.8%), and by the three-month mark (T2), it had declined even further to 11.5% (IQR: 0.0–26.9%).

Beyond the reduction in central tendency, the dispersion of bleeding scores also diminished. The IQR was reduced by 55.5% between T0 and T1 and by an additional 12.7% between T1 and T2, suggesting a growing homogeneity in clinical response over time. These changes were statistically significant: the Wilcoxon signed-rank test confirmed a significant reduction in bleeding scores both between T0 and T1 (*p* < 0.001) and between T1 and T2 (*p* < 0.001). Together, these findings indicate a clear and sustained improvement in gingival health following the intervention.

### 3.3. Site-Specific Gingival Bleeding by Tooth

**Figure 3:**
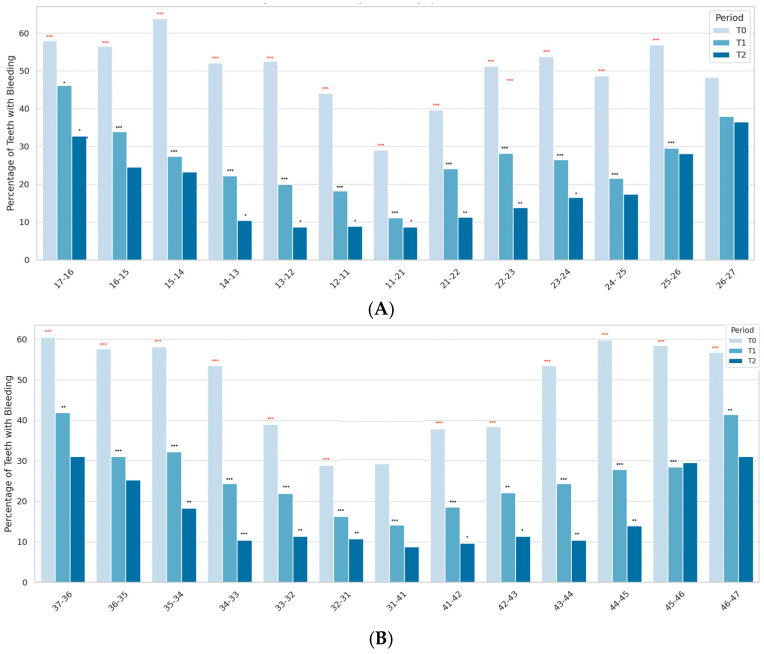
Site-Specific Changes in Gingival Bleeding (%) by Tooth Pair Over Time. (**A**). Maxilla—Gingival bleeding (%) per tooth pair at baseline (T0), 1 month (T1), and 3 months (T2). (**B**). Mandible—Gingival bleeding (%) per tooth pair at baseline (T0), 1 month (T1), and 3 months (T2). Significance levels: * *p* < 0.05; ** *p* < 0.01; *** *p* < 0.001.

Figure 3 illustrates the distribution of bleeding on probing (%) for each tooth across the three clinical visits.

The charts display the percentage of teeth with gingival bleeding at each time point (T0, T1, T2), shown as side-by-side bars for each tooth pair. A progressive reduction in bleeding was observed for most sites over time. Black asterisks above individual bars indicate the significance of pairwise comparisons between consecutive time points (T0 vs. T1, T1 vs. T2), assessed using the Wilcoxon signed-rank test: * *p* < 0.05, ** *p* < 0.01, *** *p* < 0.001. Red asterisks above bar groups indicate significant differences between T0 and T2: *** *p* < 0.001.

A consistent decrease in bleeding was observed across nearly all sites over time, with the most pronounced reductions occurring in posterior regions. At baseline (T0), the highest bleeding prevalence was recorded in posterior sites, particularly molars such as 17-16 and 26-27, with values exceeding 80%. In contrast, anterior teeth showed significantly lower bleeding levels, often below 30%. Premolars exhibited intermediate values, reflecting their transitional anatomical position and access complexity.

By T1, significant decreases were observed across most tooth pairs, including molars and premolars. These improvements were further enhanced at T2, with the most substantial reductions occurring on molars. For example, bleeding levels dropped from 85.7% to 12.5% for 17-16 (*** *p* < 0.001) and from 82.1% to 10.7% for 26-27 (*** *p* < 0.001). Anterior teeth (e.g., 11-21, 31-41) reached minimal bleeding values at T2, generally below 5%.

Statistical comparisons confirmed significant reductions between T0 and T2 for the majority of tooth pairs, especially on molars. Bilateral symmetry was observed, suggesting homogeneous improvements across right and left quadrants.

### 3.4. Multivariate Analysis of Factors Associated with Gingival Bleeding at Baseline

**Figure 4:**
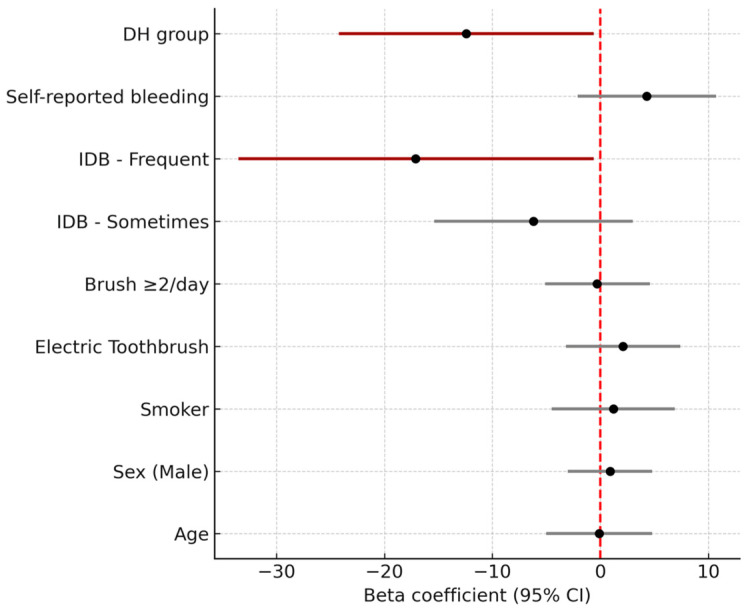
Multivariate linear regression model assessing factors associated with gingival bleeding at baseline.

A multiple linear regression analysis was conducted to explore factors independently associated with gingival bleeding at the baseline visit (Figure 4). The model included the following covariates: age, sex, smoking status, type of toothbrush (manual vs. electric), toothbrushing frequency, interdental cleaning habits, self-reported gingival bleeding, and professional group (DH: Dental Hygienists; DS: Dental Students, including Master students).

Forest plot displaying β coefficients and 95% confidence intervals (CI) from the linear regression model predicting the percentage of sites with gingival bleeding. Variables include age, sex, smoking status, toothbrush type (manual vs. electric), brushing frequency, interdental brushing frequency, self-reported bleeding, and student group (dental hygiene vs. dental students). Red bars indicate statistically significant predictors (*p* < 0.05). Frequent use of interdental brushes and being in the dental hygiene (DH) group were significantly associated with lower bleeding scores. The dashed vertical line marks the null effect (β = 0). The x-axis has been centered on β = 0 to improve interpretability and address previous display inconsistencies.

Frequent use of interdental brushes was significantly associated with lower bleeding scores (β = −17.1; 95% CI: −33.5 to −0.6; *p* = 0.042), as was being in the dental hygiene (DH) group compared to dental students (DS) (β = −12.4; 95% CI: −24.2 to −0.6; *p* = 0.039). No other variables—including age, sex, smoking status, toothbrush type, or brushing frequency—showed statistically significant associations in the adjusted model.

### 3.5. Correlation Between Gingival Bleeding and Clinical/Behavioral Variables

**Figure 5:**
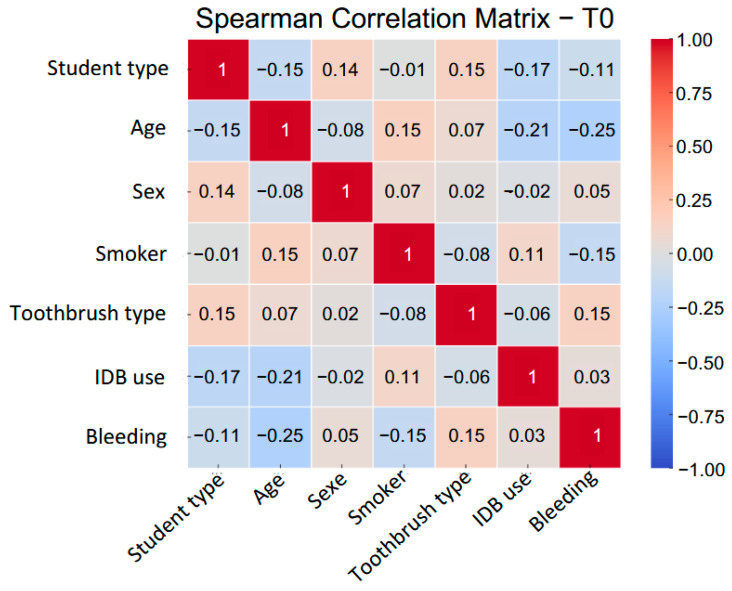

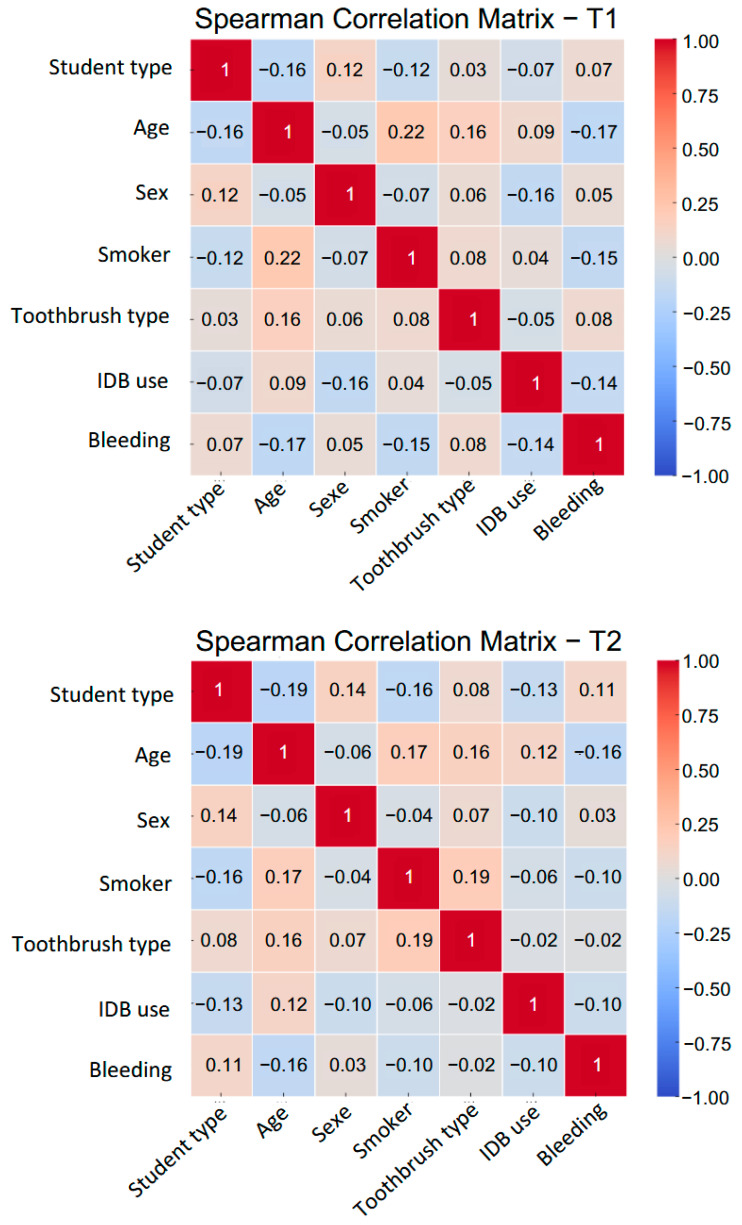
Spearman’s rank correlation matrices between bleeding scores and selected clinical or behavioral variables at each visit (T0, T1, T2).

To explore the associations between gingival bleeding and relevant clinical or behavioral factors, Spearman’s rank correlation coefficients were calculated at each time point (T0, T1, T2). As shown in Figure 5, most associations were weak and varied over time, underscoring the multifactorial nature of gingival inflammation.

At baseline (T0), bleeding scores showed a modest positive correlation with electric toothbrush use (ρ = +0.19) and weak negative correlations with age (ρ = −0.17) and tobacco use (ρ = −0.13). These associations likely reflect differences in baseline behaviors.

At T1, a negative correlation emerged between interdental brush (IDB) use and bleeding (ρ = −0.14), suggesting an early clinical response. However, this association was no longer apparent at T2 (ρ = +0.01), possibly reflecting lower adherence or a stabilization of clinical outcomes.

The correlation between electric toothbrush use and bleeding also changed over time, reversing direction from T0 to T2. This likely reflects behavioral changes rather than a direct effect of brushing mode, as conventional brushing does not address interdental inflammation effectively.

Overall, the weak and inconsistent correlations observed across visits highlight the limitations of bivariate analyses and support the use of multivariate longitudinal models to better understand the clinical effects of interdental hygiene education.

Correlation coefficients (Spearman’s ρ) are shown for all variables, numerically coded where needed. “UNIV” = study center (Pisa, Rome, Ancona); “STUDENT TYPE” = student group (dental students or dental hygienists); “AGE” = age in years; “SEX (M = 1)” = male; “SMOKER (Yes = 1)” = smoker; “TOOTHBRUSH TYPE (Electric = 1)” = electric brush; “IDB USE” = frequency of interdental brushing; “BLEEDING” = percentage of interdental sites with bleeding. Color intensity reflects the strength and direction of correlation. These matrices illustrate the evolving relationships between gingival bleeding and behavioral, demographic, and contextual variables across time points (T0, T1, T2).

**Figure 6:**
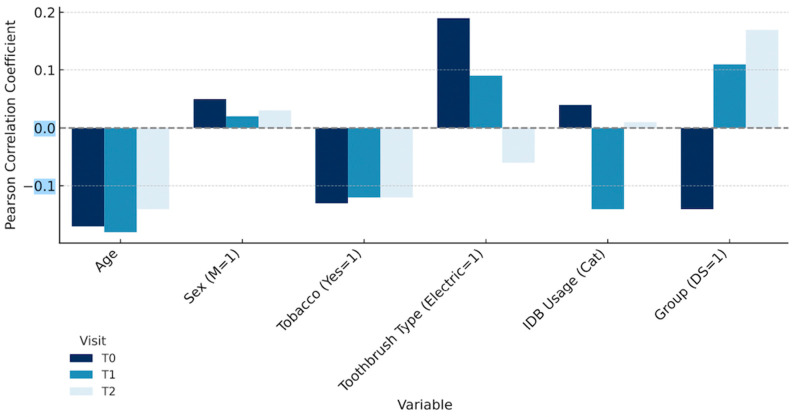
Temporal variation in Spearman correlation coefficients between bleeding scores and selected clinical/behavioral variables (T0–T2).

To further explore the relationship between behavioral or clinical characteristics and gingival bleeding over time, we examined Spearman’s rank correlation coefficients at each visit (Figure 6). Most associations were weak (r < 0.20) and inconsistent across time points.

Bar plots represent the strength and direction of Spearman’s rank correlations (ρ) between the percentage of sites with gingival bleeding and key covariates at baseline (T0), 1 month (T1), and 3 months (T2). Most associations were weak and varied over time, with no consistent predictive factor across visits. These findings illustrate the limited explanatory power of isolated behavioral indicators and support the use of adjusted longitudinal models to interpret intervention outcomes.

Most associations were weak (ρ < 0.20) and inconsistent across time points. At baseline (T0), bleeding showed a weak positive correlation with electric toothbrush use (ρ = +0.19) and weak negative correlations with age (ρ = −0.16) and tobacco use (ρ = −0.13). IDB usage at T0 was nearly uncorrelated (ρ ≈ 0.00). At T1, following the introduction of calibrated interdental brushes, IDB use showed a weak negative correlation with bleeding (ρ = −0.14), suggesting a potential early benefit. This association, however, disappeared by T2 (ρ = +0.01), possibly due to reduced adherence or a ceiling effect.

Similarly, the correlation with toothbrush type shifted from positive at T0 (ρ = +0.19) to slightly negative at T2 (ρ = −0.07), reinforcing the idea that brushing mode (manual vs. electric) has limited clinical relevance in the absence of interdental hygiene.

As illustrated, the strength and direction of associations between bleeding scores and individual covariates (age, tobacco use, toothbrush type, and IDB usage) remained unstable and weak across time points, highlighting the complex and multifactorial nature of gingival inflammation in educational settings.

### 3.6. Adjusted Longitudinal Analysis

To assess the effect of interdental brush usage on the progression of gingival bleeding, we performed a longitudinal multivariate analysis using Generalized Estimating Equations (GEE). This method accounts for within-subject correlation and is well suited for non-normally distributed repeated measures data. The model included time (T0, T1, T2), IDB usage group, and their interaction, adjusting for baseline bleeding, age, sex, smoking status, toothbrush type, and student group.

Bar heights represent estimated mean bleeding scores (%) adjusted for baseline bleeding (T0), age, sex, smoking status, toothbrush type, student group, and study center (Rome, Ancona, Pisa). Error bars indicate 95% confidence intervals derived from the Generalized Estimating Equations (GEE) model. Red asterisks (*) denote statistically significant differences between IDB usage groups at each time point (* *p* < 0.05). In some cases, confidence intervals extend beyond the top of the bars; this reflects statistical uncertainty and does not affect the validity of the estimates.

The variable center was included in the adjusted model to control for institutional or regional influences. Its effect was not statistically significant overall, although a modest trend was observed for participants from Rome, who showed slightly higher bleeding scores at baseline. These differences did not persist after adjustment and did not alter the overall findings.

**Figure 7:**
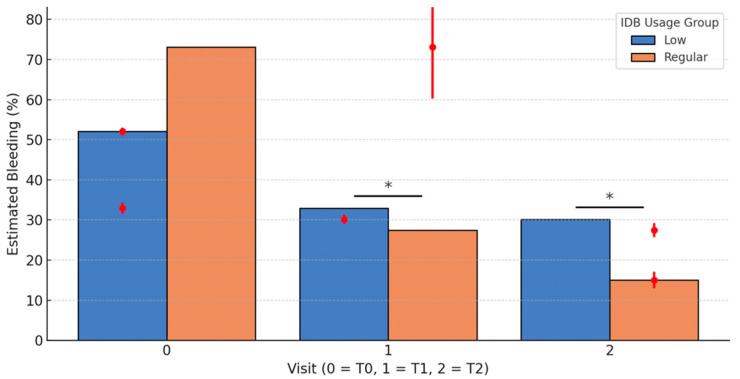
Predicted bleeding levels by interdental brush (IDB) usage group at follow-up visits (T1 and T2), based on GEE modeling. Significance level: * *p* < 0.05.

Compared to low users, regular users exhibited significantly lower predicted bleeding scores at both T1 (mean difference: −16.1 percentage points; *p* < 0.001) and T2 (−14.5 points; *p* < 0.001), indicating a sustained benefit. Among the covariates, age was significantly associated with reduced bleeding at T1 (β = −0.62; *p* = 0.011), but not at T2 (*p* = 0.50). These results are illustrated in Figure 7, which displays estimated marginal means of bleeding over time by usage group. The GEE model confirms a consistent and independent association between regular interdental brushing and improved gingival outcomes.

### 3.7. Gingival Bleeding Progression According to Interdental Brush Usage

**Figure 8:**
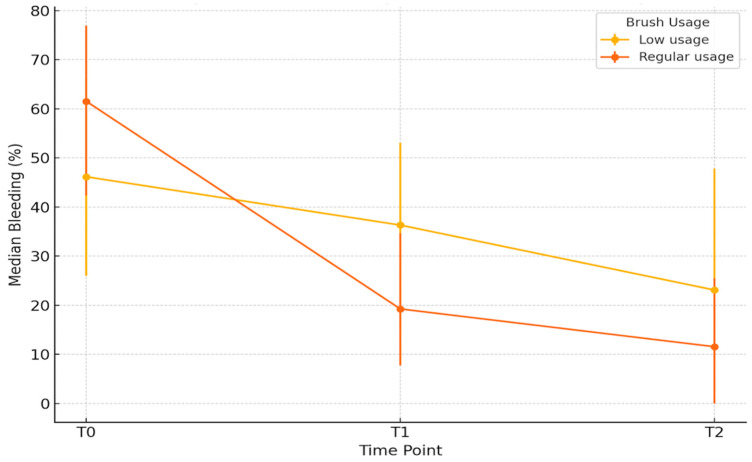
Median bleeding (%) over time by interdental brush usage (with IQR).

Initial descriptive analysis revealed a marked difference in median gingival bleeding reduction between groups stratified by interdental brush usage frequency (Figure 8). Between baseline (T0) and 1 month (T1), the median reduction in bleeding was −3.8% [IQR: 17.2] for low users, compared to −38.3% [IQR: 35.4] for regular users (*p* < 0.001). Between T0 and 3 months (T2), median reductions were −8.0% [IQR: 30.8] and −46.2% [IQR: 38.5], respectively (*p* < 0.001). Furthermore, from T1 to T2, regular users continued to improve (median Δ = −7.7% [IQR: 15.4]), whereas low users showed minimal change (median Δ = −0.3% [IQR: 10.3]; *p* = 0.0036).

Participants with higher baseline bleeding (T0) were more likely to adopt regular interdental brush use at T1 and T2. This behavioral association highlights the potential for reverse causality, where increased bleeding prompts greater interdental cleaning frequency. However, the longitudinal analyses adjusted for baseline bleeding (ANCOVA) confirmed that regular brush use was independently associated with a significant and sustained reduction in bleeding over time.

To better isolate the specific effect of interdental brush use, an adjusted analysis using ANCOVA was conducted, controlling for baseline bleeding scores, age, sex, smoking status, toothbrush type, and student group. The predicted bleeding scores from this model indicate that at T1, regular users had significantly lower predicted bleeding compared to low users (mean difference: −16.3 percentage points; *p* < 0.001). This benefit persisted at T2 with a mean difference of −14.9 points (*p* < 0.001). Age was also significantly associated with reduced bleeding at T1 (β = −0.63; *p* = 0.010), but this association was not maintained at T2 (*p* = 0.53). Some predicted values slightly below zero reflect a known artifact of the linear regression model and do not have clinical relevance.

### 3.8. Subgroup Analysis of Interaction Effects

**Figure 9:**
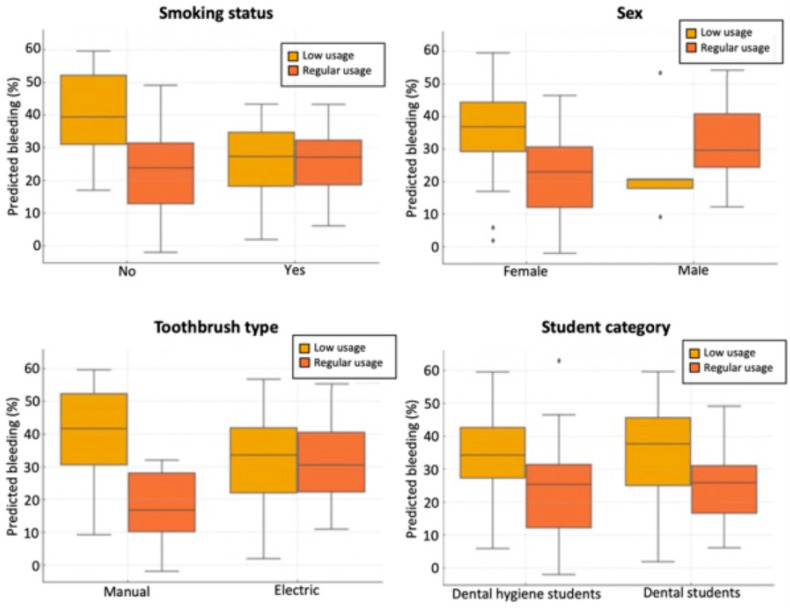
Predicted gingival bleeding at T1 according to interdental cleaning usage, stratified by smoking status, sex, toothbrush type, and student category.

We investigated whether the effect of regular interdental brush use on gingival bleeding at T1 varied across key subgroups, including smoking status, sex, toothbrush type, and student type. These interaction effects are illustrated in Figure 9, which presents predicted bleeding levels stratified by interdental brush use within each subgroup.

A significant interaction was observed with smoking status (*p* = 0.014). Among non-smokers, regular users exhibited markedly lower predicted bleeding (median: 10.1%, IQR: 20.5) compared to low users (median: 27.0%, IQR: 24.6; *p* < 0.001). In contrast, smokers showed a smaller difference between regular and low users (24.7% vs. 32.5%, respectively; *p* = 0.021), indicating that smoking attenuates the beneficial effect of regular interdental brushing.

A similar pattern was found for toothbrush type (*p* = 0.002 for interaction). Among manual toothbrush users, regular interdental brushing was associated with a substantial reduction in predicted bleeding (median: 11.8%, IQR: 21.1) versus low usage (29.1%, IQR: 26.4; *p* < 0.001). Among electric toothbrush users, the reduction was less pronounced, though still significant (17.6% vs. 26.8%; *p* = 0.037).

The interaction with sex approached statistical significance (*p* = 0.069), suggesting a possible trend towards greater benefit in females, although this did not reach the conventional threshold. No significant interaction was observed for student type (*p* = 0.288), indicating consistent effects of interdental brush use across educational backgrounds.

## 4. Discussion

This prospective cohort study demonstrated that three months of daily use of calibrated interdental brushes produced a rapid, substantial, and sustained reduction in gingival bleeding among dental and dental hygiene students. At baseline, approximately half of all interdental sites per participant exhibited bleeding upon brushing, despite the generally healthy status and relatively high oral health awareness in this population. This observation highlights the limitations of conventional brushing and the importance of interdental biofilm disruption, even among young individuals.

Although interdental brushing has been previously investigated by our group in other populations [8,19], the present study differs in several important aspects. First, it focuses on a specific academic population, dental and dental hygiene students, who represent future oral health professionals and play a pivotal role in modeling preventive behaviors. Second, this is the first time that a harmonized, multicentric educational protocol has been evaluated longitudinally across multiple academic centers, allowing both behavioral and clinical outcomes to be examined in a controlled pedagogical context. Third, we applied an advanced multivariable longitudinal approach (GEE) to assess the effects of interdental brushing over time, accounting for repeated measures, center effects, and potential confounders. These features differentiate the present study from prior work and provide novel insights into educational strategies for oral health promotion.

The impact of the intervention was both immediate and progressive. After just one month of regular interdental brushing, the median bleeding score dropped dramatically from 50.0% to 15.4% and further to 11.5% at three months (*p* < 0.001). These findings indicate that daily calibrated interdental hygiene significantly improves gingival health in a relatively short time frame. The continued reduction between one and three months suggests not only initial clinical benefit but also potential for sustained improvement with maintained behavior.

Regular users of interdental brushes—those who reported daily or near-daily use—experienced the greatest clinical benefit, with reductions in bleeding exceeding 15 to 16 percentage points compared to infrequent users at both one and three months (*p* < 0.001). Conversely, those who used interdental brushes irregularly showed only marginal improvements after the first month, underscoring the importance of consistency in achieving optimal outcomes.

Interestingly, differences were observed between subgroups at baseline: dental hygiene (DH) students exhibited significantly lower bleeding scores than dental students (DS), and students who already practiced interdental cleaning had better baseline scores. These differences likely reflect the greater emphasis on preventive care and oral hygiene training in DH curricula. However, both DS and DH students benefited comparably from the intervention, with no significant interaction between student group and outcome trajectory. This suggests that even students with limited prior experience or motivation can improve their gingival health when exposed to structured training and calibrated tools.

These findings are consistent with, and extend, prior studies on interdental hygiene. Bourgeois et al. [19] reported a 72% reduction in interdental bleeding frequency after three months of daily use of calibrated interdental brushes—a result closely mirroring the 70–80% relative reduction observed in our cohort. The early improvements reported in their study (noted after just one week) align with our one-month follow-up, indicating that the clinical response to daily interdental brushing is both rapid and robust. Both studies also observed that anterior interdental sites responded more quickly than posterior ones, which is plausible given anatomical constraints and brush access. In our sample, posterior molar regions still exhibited residual bleeding (~10–13%) at three months, suggesting that additional instruction or adjustment of brush diameter may be needed to optimize outcomes in these areas.

A split-mouth randomized controlled trial by Bourgeois et al. [8] found an 85% decrease in bleeding incidence with calibrated interdental brushing compared to a 27% reduction in control sites without interdental cleaning. The magnitude of the difference further confirms the efficacy of interdental cleaning and supports the conclusion that clinical improvement is attributable to the mechanical disruption of biofilm rather than external behavioral influences alone. Reductions in pathogenic bacterial loads observed in the same study add a microbiological dimension to our clinical findings, reinforcing the rationale for interdental prophylaxis.

Furthermore, Carrouel et al. [20] demonstrated that even periodontally healthy young adults may harbor significant levels of pathogens such as Streptococcus mutans and members of the “red complex” in their interdental spaces. This microbial load, invisible to patients and undetected in conventional exams, underscores the clinical relevance of routine interdental cleaning [21]. While not directly assessed here, the observed improvements could plausibly be linked to the disruption of interdental plaque, which is known to harbor pathogenic biofilms contributing to gingival inflammation.

From a behavioral and clinical perspective, regular interdental brush use emerged as a strong determinant of improved gingival health. The reduction in bleeding was most pronounced among students who adhered strictly to daily use, highlighting that frequency and technique matter. This reinforces the notion that interdental hygiene should not be viewed as optional or supplementary but rather as a core component of effective oral hygiene. Clinicians should prioritize individualized instruction, including brush calibration and technique, and allocate sufficient chairside time to build patient competence and confidence.

Educational implications also emerged clearly. Dental hygiene students demonstrated better baseline gingival health, likely due to greater emphasis on prevention in their training. Yet dental students, despite less experience, achieved comparable improvements when provided with the same intervention. These findings support the value of early integration of calibrated interdental hygiene education in all dental curricula—not only to improve student health, but also to prepare future practitioners to deliver effective preventive care. Interdental prophylaxis should be presented as an essential rather than optional component of oral health instruction.

Anatomical complexity in molar regions, particularly the presence of the non-keratinized interdental col, likely explains persistent inflammation even in the context of improved overall hygiene. These areas may require enhanced instruction, specific brush sizing, or adjunctive tools to achieve optimal results. Students and patients should be made aware of these anatomical challenges and trained accordingly.

In public health terms, the rapid clinical gains observed with daily interdental brushing support efforts to promote such practices from adolescence onward. Integrating interdental cleaning into national prevention strategies and facilitating access to appropriate brushes—particularly for underserved populations—could contribute meaningfully to reducing oral health disparities. As highlighted in Bourgeois et al. [10], education-based interventions can yield rapid behavioral change and clinical improvement [22].

Understanding the evolution of behavioral adherence over time offers valuable insight. Initially, strong associations were observed between behavior and outcome, but these diminished at the three-month point. This could reflect waning adherence, a common finding in preventive behavior change, ceiling effects among those with early improvement, or complex multifactorial influences [23]. It is also likely shaped by the context of dental education. Structured training, frequent reinforcement, and supervision, as in our setting, are known to enhance student confidence and preparedness [24], which likely contributed to improved compliance. These effects, while beneficial, also limit generalizability to broader, unsupervised populations.

Adjusted longitudinal models confirmed that regular interdental brushing produced significant bleeding reductions, greater than 14 percentage points, at both one and three months. These results are consistent with previous work showing that even modest behavioral improvements, if maintained, can yield measurable clinical benefits [25] and further validate the integration of interdental hygiene into early education and patient care [26].

Subgroup analyses showed attenuated effects among smokers and electric toothbrush users. The former likely reflects reduced healing potential and immune modulation, while the latter may be due to differing behavioral patterns or reduced motivation to adopt a second tool. However, as interdental brush use was self-selected, interpretation must remain cautious due to potential confounding by motivation and behavioral intent. This aligns with behavioral literature on periodontitis patients, which emphasizes the importance of planning and goal-setting in oral hygiene [27]. Future randomized studies are needed to clarify these subgroup patterns and to guide personalized prevention strategies.

The present findings open important avenues for further research. Long-term sustainability remains a key question [28]. Follow-up periods beyond 12 months are needed to determine whether clinical benefits persist after the intervention phase ends [29]. It would be particularly valuable to examine whether initial bleeding reduction predicts continued motivation or whether personality traits and behavioral profiles can forecast adherence. Targeted interventions, such as reminder apps, structured reinforcement, or integration into periodic dental checkups, may be useful strategies to maintain behavior over time [30,31].

Although interdental brushes are more effective than floss in many studies, direct head-to-head randomized trials comparing calibrated brushing, floss, and water flossers within similar populations are still lacking. Slot et al. [32] identified this gap, calling for robust trials with standardized methodology and extended follow-up. In the meantime, Bayesian network meta-analyses such as Liang et al. [33] may help guide clinical decision-making, although their strength depends on the quality and consistency of available data.

From an educational standpoint, broader implementation of interdental hygiene curricula remains a priority. Bourgeois et al. [10,34] showed significant improvements in knowledge and behavior after targeted education. The critical question now is whether these improvements endure. Longitudinal studies tracking students into their clinical careers could assess whether intensive hygiene training leads to more consistent patient education and better long-term oral health outcomes [21].

Despite the strengths of this study—including multicenter design, standardized calibration, and detailed behavioral data—several limitations must be acknowledged. The lack of a control group and randomization, as well as the absence of a comparator group using other interdental cleaning tools, limits causal inference. The follow-up period was relatively short. Bleeding was assessed clinically, but adherence was self-reported, introducing potential bias. Also, broader health or dietary variables were not controlled. Finally, the sample was composed of students in a highly structured academic environment, which may have enhanced adherence and limits generalizability. Nonetheless, the magnitude of observed effects, multivariate adjustment, and alignment with previous trials lend credibility to the findings.

## 5. Conclusions

This study offers encouraging evidence that daily use of calibrated interdental brushes can produce rapid and meaningful improvements in gingival health, even over a relatively short period. Among dental and dental hygiene students, consistent interdental cleaning led to substantial reductions in bleeding, highlighting the clinical effectiveness of this simple yet often overlooked preventive measure.

## Data Availability

The datasets generated during and/or analyzed during the current study are available from the corresponding author on reasonable request.

## Supplementary Materials

The following supporting information can be downloaded at: https://www.mdpi.com/article/10.3390/jcm14165738/s1. Table S1: Foundational Phases: Knowledge Acquisition and Preclinical Preparation Adapted from: Bourgeois D, et al. [10]; Table S2: Clinical Skill Development. Adapted from: Bourgeois D, et al. [10]; Table S3: Supervision and Longitudinal Evaluation. Adapted from: Bourgeois D, et al. [10].

## References

[1] Herrera D., Meyle J., Renvert S., Jin L. White paper on prevention and management of periodontal diseases for oral health and general health. *FDI World Dent. Fed.*
**2018**, 2020–2011. https://www.fdiworlddental.org/resource/white-paper-prevention-and-management-periodontal-diseases-oral-health-and-general-health.

[2] Sanz M., Herrera D., Kebschull M., Chapple I., Jepsen S., Berglundh T., Sculean A., Tonetti M.S., Consultants E.W.P.a.M., Merete Aass A. (2020). Treatment of stage I–III periodontitis—The EFP S3 level clinical practice guideline. J. Clin. Periodontol..

[3] Bourgeois D. (2023). Next preventive strategies for oral health: Evolution or revolution?. Front. Public Health.

[4] Carrouel F., Viennot S., Santamaria J., Veber P., Bourgeois D. (2016). Quantitative molecular detection of 19 major pathogens in the interdental biofilm of periodontally healthy young adults. Front. Microbiol..

[5] Kotsakis G.A., Lian Q., Ioannou A.L., Michalowicz B.S., John M.T., Chu H. (2018). A network meta-analysis of interproximal oral hygiene methods in the reduction of clinical indices of inflammation. J. Periodontol..

[6] Staehle H.J., Sekundo C. (2025). History of Interdental Brushes: Origins, Developments, Perspectives. Oral Health Prev. Dent..

[7] Graziani F., Palazzolo A., Gennai S., Karapetsa D., Giuca M., Cei S., Filice N., Petrini M., Nisi M. (2018). Interdental plaque reduction after use of different devices in young subjects with intact papilla: A randomized clinical trial. Int. J. Dent. Hyg..

[8] Bourgeois D., Bravo M., Llodra J.-C., Inquimbert C., Viennot S., Dussart C., Carrouel F. (2019). Calibrated interdental brushing for the prevention of periodontal pathogens infection in young adults-a randomized controlled clinical trial. Sci. Rep..

[9] Sälzer S., Graetz C., Dörfer C.E., Slot D.E., Van der Weijden F.A. (2020). Contemporary practices for mechanical oral hygiene to prevent periodontal disease. Periodontology 2000.

[10] Bourgeois D., Saliasi I., Dussart C., Llodra J.C., Tardivo D., Laforest L., Bravo M., Viennot S., Foti B., Carrouel F. (2018). Educational outcomes of a new curriculum on interproximal oral prophylaxis for dental students. PLoS ONE.

[11] Pisano M., Giordano F., Sangiovanni G., Capuano N., Acerra A., D’Ambrosio F. (2023). The interaction between the oral microbiome and systemic diseases: A narrative review. J. Clin. Med..

[12] Bourgeois D., Weiler D., Carrouel F. (2017). Oral microbiota, intestinal microbiota and inflammatory bowel diseases. Res. Rev. Biosci..

[13] Lee Y.-H., Chung S.W., Auh Q.-S., Hong S.-J., Lee Y.-A., Jung J., Lee G.-J., Park H.J., Shin S.-I., Hong J.-Y. (2021). Progress in oral microbiome related to oral and systemic diseases: An update. Diagnostics.

[14] Bourgeois D., Inquimbert C., Ottolenghi L., Carrouel F. (2019). Periodontal pathogens as risk factors of cardiovascular diseases, diabetes, rheumatoid arthritis, cancer, and chronic obstructive pulmonary disease—Is there cause for consideration?. Microorganisms.

[15] Radaic A., Kapila Y.L. (2021). The oralome and its dysbiosis: New insights into oral microbiome-host interactions. Comput. Struct. Biotechnol. J..

[16] Azzolino D., Felicetti A., Santacroce L., Lucchi T., Garcia-Godoy F., Passarelli P.C. (2025). The emerging role of oral microbiota: A key driver of oral and systemic health. Am. J. Dent..

[17] Preshaw P.M., Ramseier C.A., Loos B.G., Balčiūnaitė A., Crnić T., Davey K., Dommisch H., Ettmayer J.B., Roberts A., Verheijck E.E. (2024). Contemporary educational methods in periodontology. J. Clin. Periodontol..

[18] Simon F., Szabó G., Orsós M., Mijiritsky E., Németh O. (2024). The Effectiveness of Individualized Oral Hygiene Education in Preventing Dental Diseases: A Clinical Study. J. Clin. Med..

[19] Bourgeois D., Saliasi I., Llodra J.C., Bravo M., Viennot S., Carrouel F. (2016). Efficacy of interdental calibrated brushes on bleeding reduction in adults: A 3-month randomized controlled clinical trial. Eur. J. Oral Sci..

[20] Carrouel F., Llodra J.C., Viennot S., Santamaria J., Bravo M., Bourgeois D. (2016). Access to interdental brushing in periodontal healthy young adults: A cross-sectional study. PLoS ONE.

[21] Bourgeois D., Gonçalves L.S., Lima-Junior J.d.C., Carrouel F. (2022). The Oral Microbiome is a Key Factor in Oral and Systemic Health.

[22] Bourgeois D.M., Phantumvanit P., Llodra J.C., Horn V., Carlile M., Eiselé J.L. (2014). Rationale for the prevention of oral diseases in primary health care: An international collaborative study in oral health education. Int. Dent. J..

[23] Hajishengallis G., Chavakis T. (2021). Local and systemic mechanisms linking periodontal disease and inflammatory comorbidities. Nat. Rev. Immunol..

[24] Jankowski J., Krokosz S., Zięba S., Komandera D., Kochańska B., Fiegler-Rudol J., Zawilska A., Feret R., Szczeklik K., Gliwa K. (2025). Self-assessed preparedness of final-year dental students and dental interns in Poland: A multi-institutional study. BMC Med. Educ..

[25] Deeg I., Wicht M., Barbe A., Derman S. (2024). Self-determined use of provided powered oral hygiene devices leads to improved gingival health after 1 year: A longitudinal clinical trial. BMC Oral Health.

[26] Dimenäs S.L., Jönsson B., Andersson J.S., Lundgren J., Petzold M., Abrahamsson I., Abrahamsson K.H. (2022). A person-centred, theory-based, behavioural intervention programme for improved oral hygiene in adolescents: A randomized clinical field study. J. Clin. Periodontol..

[27] Vilar Doceda M., Petit C., Huck O. (2023). Behavioral interventions on periodontitis patients to improve oral hygiene: A systematic review. J. Clin. Med..

[28] Salhi L., De Carvalho B., Reners M. (2022). Update on the roles of oral hygiene and plaque control on periodontal disease. Periodontitis Adv. Exp. Res..

[29] Holtfreter B., Conrad E., Kocher T., Baumeister S.E., Völzke H., Welk A. (2024). Interdental cleaning aids are beneficial for oral health at 7-year follow-up: Results from the Study of Health in Pomerania (SHIP-TREND). J. Clin. Periodontol..

[30] Clément C., Lvovschi V.-E., Verot E., du Sartz de Vigneulles B., Darlington-Bernard A., Bourgeois D., Lamure M., Vitiello F., Dussart C., Carrouel F. (2023). Supporting health education policies: Translation, cross-cultural adaptation and validation of a health literacy instrument, in French. Front. Public Health.

[31] Abbinante A., Antonacci A., Antonioni M., Butera A., Castaldi M., Cotellessa S., Di Marco C., Gangale M., Izzetti R., Luperini M. (2024). Concordance and Clinical Outcomes Improvement Following Oral Hygiene Motivation: A Systematic Review and Report of the Workshop of the Italian Societies of Dental Hygiene. Int. J. Dent..

[32] Slot D.E., Valkenburg C., Van der Weijden G. (2020). Mechanical plaque removal of periodontal maintenance patients: A systematic review and network meta-analysis. J. Clin. Periodontol..

[33] Liang M., Lian Q., Kotsakis G.A., Michalowicz B.S., John M.T., Chu H. (2020). Bayesian network meta-analysis of multiple outcomes in dental research. J. Evid. Based Dent. Pract..

[34] Bourgeois D., David A., Inquimbert C., Tramini P., Molinari N., Carrouel F. (2017). Quantification of carious pathogens in the interdental microbiota of young caries-free adults. PLoS ONE.

