# Supplementary material for: Improved Plaque-Induced Gingivitis in Students Using Calibrated Interdental Brushes: Results of a 3-Month Multicenter Educational Intervention
Source: J Clin Med. 2025 Aug 14;14(16):5738. doi: 10.3390/jcm14165738 (PMC12386892; doi:10.3390/jcm14165738)
Supplement: Supplementary file 1 [file jcm-14-05738-s001.zip › jcm-3747378-supplementary.pdf]

Table S1. Foundational Phases: Knowledge Acquisition and Preclinical Preparation – Italy. Steps 1–3 of the six-step educational sequence implemented in the Italian multicenter study, including the initial amphitheater session with baseline behavioral survey and interactive lectures, baseline knowledge assessment with targeted remediation, and advanced preclinical training.

Steps	Learning Objectives	Contents
Initial interactive lecture and baseline survey (1)	Establish baseline oral hygiene habits, behaviors, and knowledge; introduce core preventive concepts. The overarching aim is to induce a sustainable change in students' oral hygiene behaviors through a simple, reproducible educational sequence.	In-person amphitheater session for all students. Online questionnaire on oral hygiene habits and behaviors completed at the start. Interactive lectures covering: (1.1) Individual prophylaxis; (1.2) Biofilm; (1.3) Disruption of biofilm on accessible surfaces; (1.4) Interdental spaces. Total duration approximately 4 hours. The session also includes a critical analysis of oral hygiene tools (toothbrushes, calibrated probes, and interdental brushes) focusing on clinical relevance, ergonomics, feasibility, and cost-effectiveness.
Baseline knowledge assessment and targeted remediation (2)	Verify immediate knowledge acquisition and address specific gaps.	Multiple-choice questionnaire (MCQ) or equivalent quiz at the end of the initial lecture. Targeted review of concepts not sufficiently mastered by students.
Advanced preclinical training session (3)	Master the correct technique for using a manual toothbrush (biofilm disruption on accessible surfaces), the calibrated IAP probe (interdental access measurement), and calibrated interdental brushes.	Students receive: IAP Curaprox® colorimetric probe, 5 calibrated CPS interdental brushes, color code, and screening cards. Practical demonstration by experienced instructors in groups of 24 students. Hands-on exercises under supervision for all three tools: toothbrushing technique training on models or peers for accessible surfaces, correct use of the calibrated IAP probe to measure interdental diameters, and selection/technique for calibrated interdental brushes. Students are encouraged to critically assess the design, ergonomics, and clinical applicability of each tool. Training was organized in subgroups ensuring a maximum ratio of 1 instructor for 8 students, with total group size not exceeding 24.

Table S2. Clinical Skill Development and Longitudinal Maintenance – Italy. Steps 4–6 of the six-step educational sequence, including the Touch-to-Teach clinical training session, end-of-module teaching evaluation, and long-term recall with behavioral and clinical follow-up.

**Steps**	**Learning Objectives**	**Contents**
Clinical training session (Touch-to-Teach) (4)	Enable students to take control of their oral health by disrupting interdental biofilm and mastering prophylaxis technique.<br>Assess bleeding score and record interdental access diameter using the calibrated IAP probe.	Organized by experienced and calibrated instructors per center.<br>Interproximal Clinical Examination: classification of interdental space diameter with the calibrated IAP probe, assessment of bleeding conditions.<br>Touch-to-Teach method with role rotation (patient/operator/assistant).<br>Recording chart: interdental diameter and bleeding score per site. This step also reinforces correct toothbrushing technique for accessible surfaces, and provides opportunities to critically evaluate the efficiency, ergonomics, and clinical applicability of the calibrated probe and interdental brushes based on patient feedback and operator experience. Clinical training was organized in subgroups with a maximum ratio of 1 instructor for 8 students, and a total group size not exceeding 24.
End-of-module teaching evaluation (5)	Evaluate the quality of teaching delivery and formalize improvements.	Student evaluation of the module using structured feedback forms. Synthesis of corrective actions and pedagogical improvements.
Long-term recall and behavioral follow-up (6)	Maintain adherence, reinforce core clinical principles, and monitor long-term changes in behavior and bleeding scores.	Recall visits at 1 month, 3 months, and where applicable 1 year. Reinforcement of clinical fundamentals, re-motivation, re-calibration, and evaluation of behavioral and clinical outcomes over time.

Table S3. Instructor Training and Calibration – Italy. Standardized process for examiner training, inter-center calibration, and quality assurance to ensure consistent interdental space classification and bleeding assessment across study centers.

**Process**	**Objectives**	**Contents**
Centralized training and calibration	Ensure consistency in interdental space classification and bleeding assessment across centers.	16 h theoretical + practical training for all instructors.<br>Calibration on 20 volunteer students (112 sites) using IAP Curaprox® probe.<br>Minimum 85% agreement with gold standard examiner.<br>Standardized delivery of teaching content and supervision at all sites. All pedagogical materials, including lecture slides, questionnaires, and clinical instruments, were standardized and identical across all participating sites, and were derived from the French program.
